# A new type of non-Hermitian phase transition in open systems far from thermal equilibrium

**DOI:** 10.1038/s41598-021-03389-3

**Published:** 2021-12-15

**Authors:** T. T. Sergeev, A. A. Zyablovsky, E. S. Andrianov, A. A. Pukhov, Yu. E. Lozovik, A. P. Vinogradov

**Affiliations:** 1 Moscow Institute of Physics and Technology, 9 Institutskiy Per., Moscow, Russia 141700; 2 Dukhov Research Institute of Automatics (VNIIA), 22 Sushchevskaya, Moscow, Russia 127055; 3 Institute for Theoretical and Applied Electromagnetics, 13 Izhorskaya, Moscow, Russia 125412; 4 Kotelnikov Institute of Radioengineering and Electronics RAS, 11-7 Mokhovaya, Moscow, Russia 125009; 5 Institute of Spectroscopy Russian Academy of Sciences, 5 Fizicheskaya, Troitsk, Moscow, Russia 108840; 6 MIEM at National Research University Higher School of Economics, Moscow, Russia

**Keywords:** Statistical physics, thermodynamics and nonlinear dynamics, Phase transitions and critical phenomena

## Abstract

We demonstrate a new type of non-Hermitian phase transition in open systems far from thermal equilibrium, which can have place in the absence of an exceptional point. This transition takes place in coupled systems interacting with reservoirs at different temperatures. We show that the spectrum of energy flow through the system caused by the temperature gradient is determined by the $\varphi^{4}$-potential. Meanwhile, the frequency of the maximum in the spectrum plays the role of the order parameter. The phase transition manifests itself in the frequency splitting of the spectrum of energy flow at a critical point, the value of which is determined by the relaxation rates and the coupling strength. Near the critical point, fluctuations of the order parameter diverge according to a power law with the critical exponent that depends only on the ratio of reservoirs temperatures. The phase transition at the critical point has the non-equilibrium nature and leads to the change in the energy flow between the reservoirs. Our results pave the way to manipulate the heat energy transfer in the coupled out-of-equilibrium systems.

## Introduction

Non-Hermitian systems possess many unusual properties^1,2^, and one of the most interesting features of these systems is the presence of exceptional points (EPs)^3,4^. An EP is a spectral singularity in the parameter space of a non-Hermitian system in which two or more eigenstates become linearly dependent and their eigenfrequencies coalesce^2–4^. When an EP is crossed, the eigenstates of the non-Hermitian system change; for example, the symmetry of the eigenstates may be spontaneously broken^4–7^. Of the non-Hermitian systems that have EPs, PT-symmetric systems are notable^3–7^. In these systems, the EP separates areas of parameter space with PT-symmetric and non-PT-symmetric eigenstates^4–7^. Other examples of non-Hermitian systems with an EP include strongly coupled cavity-atom systems^3,8^, polariton systems^9–11^, two dimensional lattice systems^12,13^, optomechanical systems^14,15^, and even conventional laser systems^16^. Non-Hermitian systems with EPs have a number of unique properties, due to which they have found many applications^3,4,6,7^. For example, they are used to enhance the sensitivity of laser gyroscopes^17^ and sensors^18–21^, to achieve single-mode lasing in multimode systems^22,23^ and directional lasing^24^, and to control the lasing threshold^25–27^. In addition, a laser system with an EP can be used to achieve lasing with a negative population inversion^28,29^.

Due to the changes in the eigenstates of a non-Hermitian system at an EP, the transition through the EP is often associated with the point of non-Hermitian phase transition^3,4^. In experiments, non-Hermitian phase transitions are observed as frequency splitting in the system spectrum^10,11,30,31^. However, relaxation and noise tend to shift this splitting point in the spectrum away from the EP^32–35^. An analogy with a phase transition is further complicated by the fact that non-Hermitian systems are generally non-equilibrium systems. Indeed, non-Hermitian systems with EPs inevitably consist of several subsystems that interact with the different reservoirs. These reservoirs of various types (e.g., photon and phonon reservoirs) are not necessarily in thermal equilibrium with each other, which moves the system far from thermal equilibrium and results in an energy flow between the reservoirs^36–42^. These non-equilibrium systems can serve as a basis for phonons analogs of electrons and photonics devices^36–38,40,41^.

In this paper, we use the example of two coupled oscillators that interact with reservoirs at different temperatures to demonstrate a new type of non-Hermitian phase transition. This transition leads to frequency splitting in the spectrum of energy flow between the reservoirs. We show that this spectrum is determined by an analog of the $\varphi^{4}$-potential, and that it exhibits frequency splitting at a critical point (CP) above which there are two minima in the potential. Using an analogy with the theory of second-order phase transitions, we determine the frequency of the maximum in the spectrum as an order parameter for this non-Hermitian transition. We demonstrate that near the CP, fluctuations of the order parameter diverge according to a power law. The corresponding critical exponent depends only on the ratio of the temperatures of the reservoirs, and remains unchanged when the temperatures of all the reservoirs change by the same factor. This indicates the non-equilibrium nature of the phase transition at the CP. It is remarkable that although the energy flow between the reservoirs is proportional to the difference of their temperatures, the CP does not depend on the reservoir temperatures. Like an EP, the CP depends only on the relaxation rates of the system and the coupling strength between its subsystems.

Our results open the way for observing non-Hermitian phase transitions in systems far from thermal equilibrium. The conditions for a CP in this transition differ from the conditions for an EP, thus making it possible to observe non-Hermitian phase transitions in systems without an EP, e.g., in a system of two coupled oscillators with the same relaxation rate but with different reservoirs temperatures. The predicted non-Hermitian phase transition leads to the change in the energy flow between the reservoirs that paves the way to control the heat energy transfer in nanoscale and can be used to develop phonon devices^36–38,40,41^.

## Model

We consider two coupled oscillators interacting with their own reservoirs. The reservoirs are in thermodynamic equilibrium with temperatures $T_{1}$ and $T_{2}$, respectively. This system can be implemented, for example, based on optically coupled nanomechanical resonators^42^. By eliminating the degrees of freedom of the reservoir using the Born-Markovian approximation^43,44^, we can obtain the equations for the oscillator slow amplitudes^43,44^ (see also “Methods” section):

1 $$\frac{d}{dt}\left( {\begin{array}{*{20}l} {a_{1} } \\ {a_{2} } \\ \end{array} } \right) = \left( {\begin{array}{*{20}l} { - \gamma_{1} } & { - i\Omega } \\ { - i\Omega } & { - \gamma_{2} } \\ \end{array} } \right)\left( {\begin{array}{*{20}l} {a_{1} } \\ {a_{2} } \\ \end{array} } \right) + \left( {\begin{array}{*{20}l} {\xi_{1} } \\ {\xi_{2} } \\ \end{array} } \right).$$

Here, $\vec{a} = \left( {a_{1} ,a_{2} } \right)^{T}$ is a vector of the amplitudes of the first and second oscillators, respectively; $\Omega$ is the coupling strength between the oscillators; $\gamma_{1,2}$ are the relaxation rates of the oscillators; and $$\vec{\xi } = \left( {\begin{array}{*{20}l} {\xi_{1} } & {\xi_{2} } \\ \end{array} } \right)^{T}$$ is a noise term that always appears together with relaxation terms^43,44^. In the Born–Markovian approximation, the noises are delta-correlated and are connected with the relaxation rates according to^43,45^

2 $$\left\langle {\vec{\xi }} \right\rangle = 0,\,\,\,\,\,\left\langle {\vec{\xi }^{*} (t + \tau )\vec{\xi }^{T} (t)} \right\rangle = 2\hat{D}\delta (\tau ),$$

where $$\hat{D} = \left( {\begin{array}{*{20}l} {\gamma_{1} T_{1} } & 0 \\ 0 & {\gamma_{2} T_{2} } \\ \end{array} } \right)$$ is a diffusion matrix (we put $k_{{\text{B}}} = 1$). For brevity, we denote the matrix on the right-hand side of Eq. (1) as $$\hat{M} = \left( {\begin{array}{*{20}l} { - \gamma_{1} } & { - i\Omega } \\ { - i\Omega } & { - \gamma_{2} } \\ \end{array} } \right)$$.

We calculate the spectrum of stationary fluctuations (i.e., at times $t \gg \gamma_{1,2}^{ - 1}$), of the system using the Wiener–Khinchin theorem^46,47^:

3 $$\hat{S}(\omega ) = \frac{1}{2\pi }\int\limits_{ - \infty }^{ + \infty } {d\tau \exp \,( - i\omega \tau )} \left\langle {\vec{a}^{*} (\tau )\vec{a}^{T} } \right\rangle_{st} ,$$

where $\left\langle {\vec{a}^{*} (\tau )\vec{a}^{T} } \right\rangle_{st}$ is a matrix of two time correlators that is determined using regression theorem as follows (see “Methods” section for details):

$$\left\langle {\vec{a}^{*} (\tau )\,\vec{a}^{T} } \right\rangle_{st} = \left\{ {\begin{array}{*{20}c} {\exp (\hat{M}^{*} \tau )\left\langle {\vec{a}^{*} \vec{a}^{T} } \right\rangle_{st} ,\,\,\,\,\,\tau > 0} \\ {\left\langle {\vec{a}^{*} \vec{a}^{T} } \right\rangle_{st} \exp ( - \hat{M}^{T} \tau ),\,\,\,\,\,\tau < 0} \\ \end{array} } \right..$$

The averages $\left\langle {\vec{a}^{*} \vec{a}^{T} } \right\rangle_{st} = \left\langle {\vec{a}^{*} \left( {\tau = 0} \right)\vec{a}^{T} } \right\rangle_{st}$ obey the following condition^43^ (see also the “Methods” section):

4 $$\hat{M}^{*} \left\langle {\vec{a}^{*} \vec{a}^{T} } \right\rangle_{st} + \left\langle {\vec{a}^{*} \vec{a}^{T} } \right\rangle_{st} \hat{M}^{T} + 2\hat{D} = 0.$$

It should be noted that the noise results in nonzero stationary values for the oscillator energies $\left\langle {a_{1}^{ * } a_{1} } \right\rangle_{st}$ and $\left\langle {a_{2}^{ * } a_{2} } \right\rangle_{st}$ as well as the value $\left\langle {a_{1}^{ * } a_{2} } \right\rangle_{st}$. The real part, ${\text{Re}} \left\langle {a_{1}^{ * } a_{2} } \right\rangle_{st}$, represents the interaction energy between the oscillators, while the imaginary part, ${\text{Im}} \left\langle {a_{1}^{ * } a_{2} } \right\rangle_{st}$, represents the energy flow from the first oscillator to the second.

Using the Eqs. (3) and (4) we obtain the following expression for the spectrum^43^:

5 $$\hat{S}(\omega ) = \frac{1}{\pi }(\hat{M}^{*} - i\omega \hat{I})^{ - 1} \hat{D}(\hat{M}^{T} + i\omega \hat{I})^{ - 1} .$$

Using the definitions of matrices $\hat{M}$ and $\hat{D}$, we obtain:

6 $$\hat{S}(\omega ) = \frac{1/\pi }{{\Phi (\omega )}}\left( {\begin{array}{*{20}l} {\gamma_{1} T_{1} (\gamma_{2}^{2} + \omega^{2} ) + \gamma_{2} T_{2} \,\Omega^{2} } \hfill & {i\,\Omega \left( {\gamma_{2} T_{2} (\gamma_{1} + i\omega ) - \gamma_{1} T_{1} (\gamma_{2} - i\omega )} \right)} \hfill \\ {i\Omega \,\left( {\gamma_{1} T_{1} (\gamma_{2} + i\omega ) - \gamma_{2} T_{2} (\gamma_{1} - i\omega )} \right)} \hfill & {\gamma_{2} T_{2} (\gamma_{1}^{2} + \omega^{2} ) + \gamma_{1} T_{1} \,\Omega^{2} } \hfill \\ \end{array} } \right),$$

where $\Phi (\omega ) = (\gamma_{1} \gamma_{2} + \Omega^{2} )^{2} - 2(\Omega^{2} - \Omega_{cr}^{2} )\omega^{2} + \omega^{4}$ and $\Omega_{cr}^{2} = \left( {\gamma_{1}^{2} + \gamma_{2}^{2} } \right)/2$. The diagonal elements of $\hat{S}(\omega )$ determine the spectra of the fluctuations of the first and second oscillators. The non-diagonal elements determine the spectrum of interaction between the oscillators. The real part determines the spectrum of fluctuations of interaction energy, while the imaginary part determines the spectrum of energy flow from one oscillator to another.

## Spectra of oscillators

In the absence of noise, the stationary state of the system of Eq. (1) is zero. The dynamics of the system when it tends to the stationary state is determined only by the eigenvalues and eigenstates of matrix $\hat{M}$, which have the form:

7 $$\lambda_{1,2} = - \frac{{\gamma_{1} + \gamma_{2} }}{2} \pm \frac{1}{2}\sqrt {\left( {\gamma_{1} - \gamma_{2} } \right)^{2} - 4\,\Omega^{2} } ,$$

and

8 $$\vec{e}_{1,2} = \left\{ {\begin{array}{*{20}l} {\frac{i}{2\Omega }\left( {\gamma_{2} - \gamma_{1} \pm \sqrt {\left( {\gamma_{1} - \gamma_{2} } \right)^{2} - 4\,\Omega^{2} } } \right)} \\ 1 \\ \end{array} } \right\}.$$

In this system, there is an EP at which the eigenvalues are equal to each other and the eigenstates coincide. The EP arises when $\Omega = \Omega_{EP} = \left| {\gamma_{2} - \gamma_{1} } \right|/2$, and separates the weak ($\Omega < \Omega_{EP}$) and strong ($\Omega > \Omega_{EP}$) coupling regimes^3,4^.

**Figure 1:**
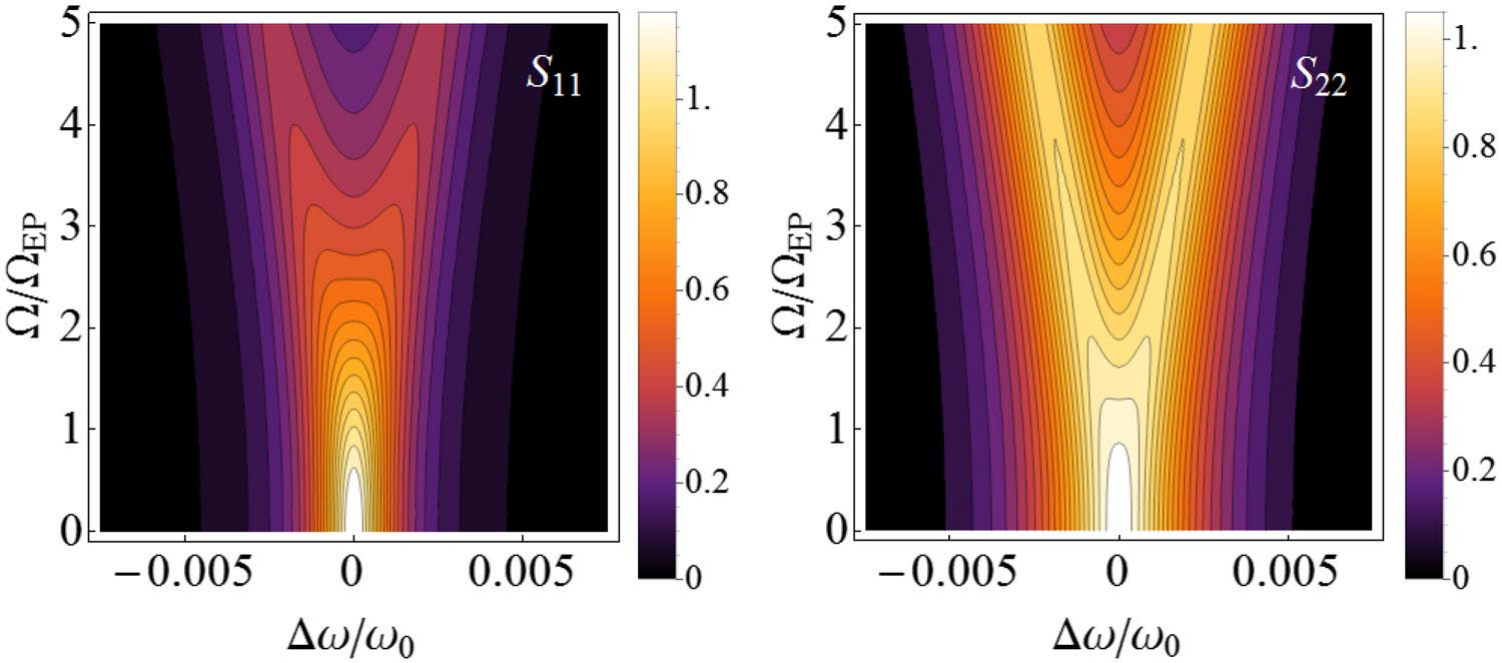
Spectra of the first and second oscillators for different values of the coupling strength $\Omega$. $\gamma_{1} = 10^{ - 3} \omega_{0}$, $\gamma_{2} = 2 \times 10^{ - 3} \omega_{0}$, $T_{1} = T_{2}$, where $\omega_{0}$ is the eigenfrequency of non-interacting oscillators, in units of which the relaxation rates and the coupling strengths are measured. Here, $\Omega_{1}^{split} \approx 2.58\,\Omega_{EP}$ and $\Omega_{2}^{split} \approx 1.12\,\Omega_{EP}$ (the analytical expressions for $\Omega_{1,2}^{split}$ are given in the Methods). The spectra of the first and second oscillators are defined by the components $S_{11}$ and $S_{22}$ of the matrix $\hat{S}(\omega )$, see Eq. (6).

**Figure 2:**
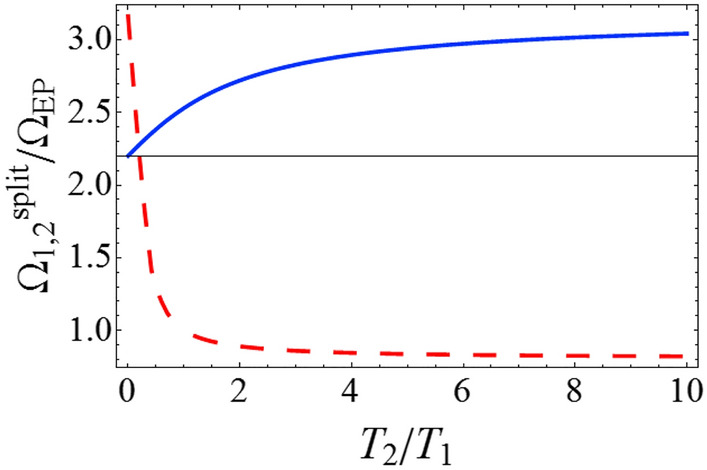
Dependence of $\Omega_{1}^{split}$ (solid blue line) and $\Omega_{2}^{split}$ (dashed red line) on the ratio of reservoir temperatures ($T_{2} /T_{1}$).

It is assumed that the transition to the strong coupling regime can be detected based on the splitting in the system spectrum^3,4^. However, splitting in the fluctuation spectra of the first and second oscillators occurs at coupling strengths of $\Omega_{1}^{split}$ and $\Omega_{2}^{split}$, which differ from the coupling strength at the EP ($\Omega_{EP}$) (Fig. 1) and from each other (the analytical formulas for $\Omega_{1,2}^{split}$ are given in the “Methods” section). That is, the splitting in the spectra of the first and second oscillators occurs at different values of the coupling strength^42^. $\Omega_{1,2}^{split}$ depend on the ratio of the reservoir temperatures ($T_{2} /T_{1}$) (Fig. 2). It should be noted that the splitting in the oscillator spectra can take place even when $\Omega < \Omega_{EP}$, i.e., in the weak coupling regime (see the dashed red line in Fig. 2).

Thus, the splitting point in the system spectrum depends on the reservoir temperatures, and generally does not coincide with the EP. We note that at this stage, it is difficult to provide a direct analogy with the standard theory of second-order phase transition, due to absence of an order parameter and the divergence of its fluctuations near the transition point. As we will see below, this analogy can be established if we consider the spectrum of the energy flow.

## Non-Hermitian phase transition in the spectrum of energy flow between the reservoirs

### Energy flow between the reservoirs

As mentioned above, there is an energy flow through the system, i.e., the system is not in thermodynamic equilibrium. The spectrum of this energy flow is determined by the product of the imaginary parts of the non-diagonal elements of the matrix $\hat{S}(\omega )$ and the coupling strength $\Omega$:

9 $$\left\langle {J\left( \omega \right)} \right\rangle = \Omega \,\,{\text{Im}} S_{12} \left( \omega \right) = - \Omega \,\,{\text{Im}} S_{21} \left( \omega \right) = \frac{1}{\pi \,\Phi (\omega )}\Omega^{2} \,\gamma_{1} \gamma_{2} (T_{2} - T_{1} ),$$

where $\Phi (\omega ) = (\gamma_{1} \gamma_{2} + \Omega^{2} )^{2} - 2(\Omega^{2} - \Omega_{cr}^{2} )\omega^{2} + \omega^{4}$ determines the frequency spectrum of $\left\langle {J\left( \omega \right)} \right\rangle$, which splits when $\Omega^{2} = \Omega_{cr}^{2} = \left( {\gamma_{1}^{2} + \gamma_{2}^{2} } \right)/2$. Indeed, the quantity $\left\langle {J\left( \omega \right)} \right\rangle$ represents the average energy flow between the reservoirs, and is always directed from a hotter to a colder reservoir (Eq. (9)). The amplitude of $\left\langle {J\left( \omega \right)} \right\rangle$ depends on the difference of the reservoir temperatures. However, the form of the spectrum $\left\langle {J\left( \omega \right)} \right\rangle$ does not depend on the reservoir temperatures, and is determined only by the denominator $\Phi (\omega )$. When $\Omega^{2} < \Omega_{cr}^{2}$, there is a single maximum in the spectrum $\left\langle {J\left( \omega \right)} \right\rangle$ at $\omega_{\max } = 0$. In the opposite case, when $\Omega^{2} > \Omega_{cr}^{2}$, there are two maxima in the spectrum $\left\langle {J\left( \omega \right)} \right\rangle$ at $\omega_{\max } = \pm \sqrt {\Omega^{2} - \Omega_{cr}^{2} }$. At $\Omega^{2} = \Omega_{cr}^{2}$, splitting arises in the spectrum of the energy flow.

**Figure 3:**
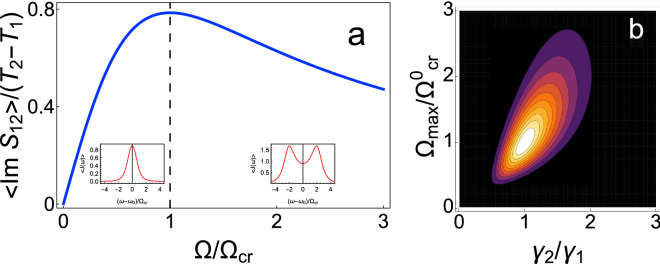
(**a**) Dependence of $\left\langle {{\text{Im}} S_{12} } \right\rangle = \int\limits_{ - \infty }^{ + \infty } {\left\langle {{\text{Im}} S_{12} \left( \omega \right)} \right\rangle d\omega }$ on the coupling strength at the condition $\gamma_{1} = \gamma_{2}$. (**b**) Dependence of $\left\langle {{\text{Im}} S_{12} } \right\rangle$ on the coupling strength and the ratio of the relaxation rates. We fix the value of $\gamma_{1}$ and change the value of $\gamma_{2}$. $\Omega_{cr}^{0} = \gamma_{2}$.

Note that the integral of imaginary part of the non-diagonal element of the matrix $\hat{S}(\omega )$ over all frequency range, $\left\langle {{\text{Im}} S_{12} } \right\rangle = \int\limits_{ - \infty }^{ + \infty } {\left\langle {{\text{Im}} S_{12} \left( \omega \right)} \right\rangle d\omega }$, depends non-monotonically on the coupling strength and the ratio of the relaxation rates, $\gamma_{2} /\gamma_{1}$. If $\gamma_{1} = \gamma_{2}$, $\left\langle {{\text{Im}} S_{12} } \right\rangle$ is maximum when $\Omega^{2} = \Omega_{cr}^{2}$, i.e., at the splitting point in the spectrum of energy flow (see Fig. 3a). This quantity reaches its absolute maximum under the condition $\gamma_{1} = \gamma_{2}$ and $\Omega^{2} = \Omega_{cr}^{2}$ (see Fig. 3b), i.e., when $\Omega = \gamma_{1} = \gamma_{2} = \Omega_{cr}^{0}$.

Note that $\Omega_{cr} \ne \Omega_{EP}$ and, moreover, at the $\Omega = \Omega_{cr}$, the eigenvalues and eigenstates of the system matrix $\hat{M}$ do not qualitatively changes. However, in the same way as $\Omega_{EP}$, $\Omega_{cr}$ does not depend on the reservoir temperatures. The expression for $\Phi (\omega )$ is an analog of the $\varphi^{4}$-potential for order parameter^48^, while $\omega_{\max }$ is an analog of an order parameter at the second-order phase transition. As we will show below, the fluctuations of $\omega_{\max }$ diverge near $\Omega_{cr}$.

### Critical behavior near the non-Hermitian transition

To establish the similarity between the non-Hermitian phase transition in a non-equilibrium system and the second-order phase transition, we study the fluctuation behavior of energy flow $J\left( \omega \right)$ near the critical coupling strength.

**Figure 4:**
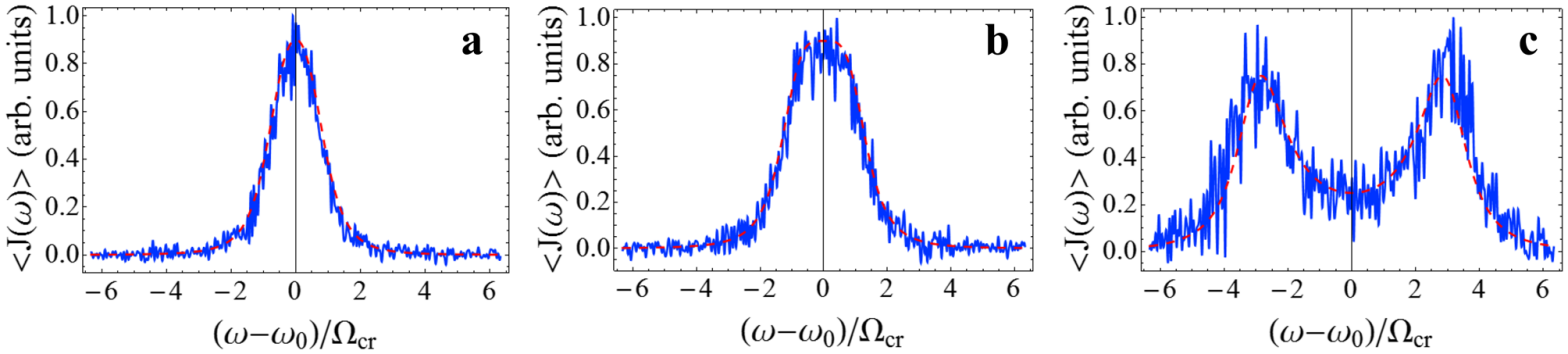
Spectra of the energy flow $\left\langle {J\left( \omega \right)} \right\rangle$, calculated by averaging over $3 \times 10^{5}$ realizations of simulations of Eq. (1) (solid blue line) and calculated using Eq. (9) (dashed red line): (**a**) $\Omega = 0.67\,\Omega_{cr}$; (**b**) $\Omega = \Omega_{cr}$; (**c**) $\Omega = 3\,\Omega_{cr}$. We put $k_{B} = 1$ thus $\left\langle {J\left( \omega \right)} \right\rangle$ is measured in units of temperature.

On average, the energy flow is directed from a hotter to a colder reservoir. The spectrum of the average energy flow, $\left\langle {J\left( \omega \right)} \right\rangle$, is determined by Eq. (9). To study the fluctuations of the energy flow, we simulate the system dynamics using Eq. (1) with noise. We calculate the stochastic time evolution of the energy flow over a finite time range $t \in \left[ {0,\,t_{f} } \right]$, and then find the Fourier spectrum of the energy flow, $J\left( \omega \right)$. For this purpose, we simulate the Eq. (1) using the Euler difference scheme that is stable near the stationary state^49,50^. The noise terms acting on the first and second oscillators are modeling as independent of each other random processes with independent increments over time. These noises are delta-correlated in time (white noise) and their average values are zero, see Eq. (2) and “Methods” section. The noise correlators are determined by diagonal elements of the diffusion matrix $\hat{D}$, which are proportional to the product of the reservoir temperature and the relaxation rate of the corresponding oscillators (see Eq. (2), “Methods” section)^50^. By averaging over a large number of simulations of Eq. (1), we obtain spectra for $\left\langle {J\left( \omega \right)} \right\rangle$ using Eq. (9) (see Fig. 4).

**Figure 5:**
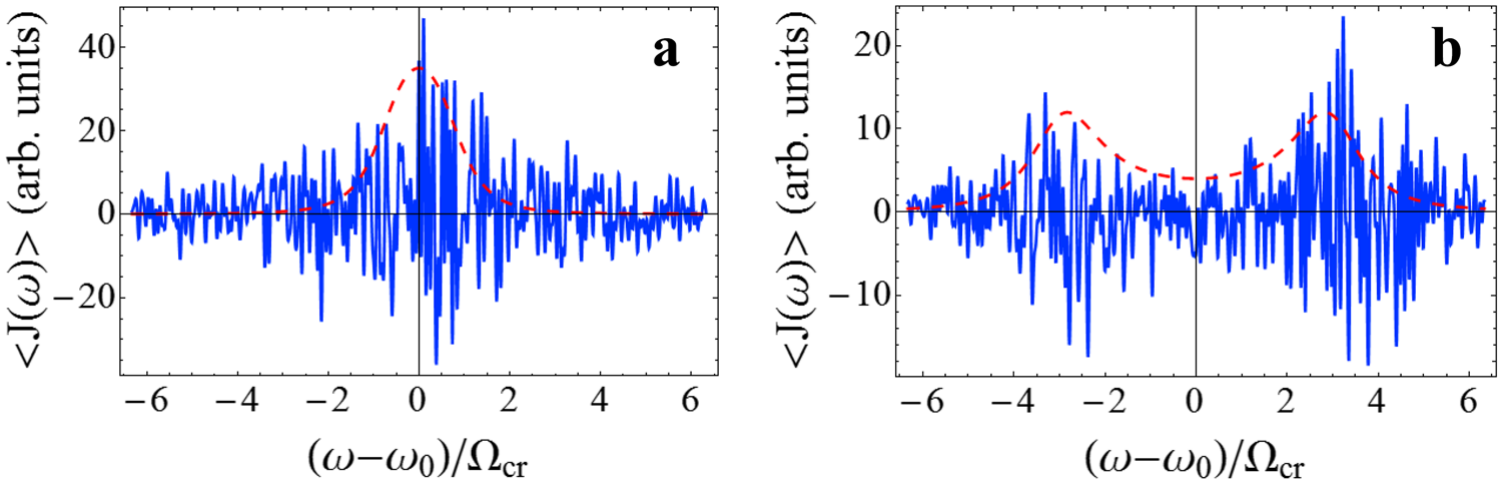
The spectra of energy flow $J\left( \omega \right)$ calculated from a single simulation of Eq. (1) (solid blue line) and the $\varphi^{4}$-potential $\Phi (\omega )$ (dashed red line) for: (**a**) $\Omega = 0.67\,\Omega_{cr}$ and (**b**) $\Omega = 3\,\Omega_{cr}$. We put $k_{B} = 1$ thus $\left\langle {J\left( \omega \right)} \right\rangle$ is measured in units of temperature.

In a given realization, the energy flow can fluctuate and the direction of flow may change. As a result, the spectrum of energy flow calculated from a single realization differs significantly from $\left\langle {J\left( \omega \right)} \right\rangle$ (Fig. 5). However, the frequency distribution of the maxima in the spectrum $J\left( \omega \right)$ resembles the form of $1/\Phi (\omega )$ (cf. the solid blue and dashed red lines in Fig. 5). That is, $\Phi (\omega )$ plays the role of a potential for the distribution of maxima in the spectrum $J\left( \omega \right)$. Accordingly, the frequency of the maximum in the spectrum $J\left( \omega \right)$ can be considered as the order parameter of the non-Hermitian phase transition in a system far from equilibrium. In turn, the splitting point in the spectrum $J\left( \omega \right)$ can be referred as a critical point (CP) of the phase transition.

**Figure 6:**
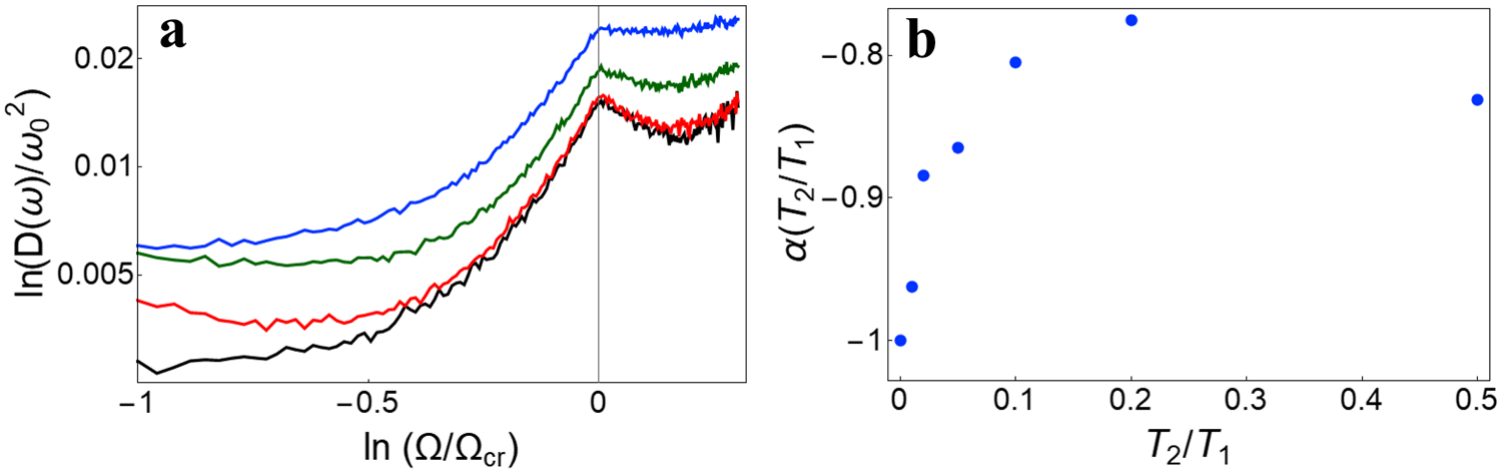
(**a**) Dependence of the dispersion $D\left( {\omega_{\max } } \right) = \left\langle {\omega_{\max }^{2} } \right\rangle - \left\langle {\omega_{\max } } \right\rangle^{2}$ in units of $\omega_{0}^{2}$ on the coupling strength for different ratios of the reservoir temperatures $T_{1,2}$: $T_{2} /T_{1} = 0$ (black line), $T_{2} /T_{1} = 0.01$ (red line), $T_{2} /T_{1} = 0.1$ (green line), $T_{2} /T_{1} = 0.5$ (blue line); (**b**) dependence of the critical exponent on the ratio of the reservoir temperatures ($T_{2} /T_{1}$).

To describe the fluctuations of the order parameter, we find the frequency of the maximum in the spectrum $J\left( \omega \right)$, i.e., $\omega_{\max }$, for each of the simulations. We then average $\omega_{\max }$ and $\omega_{\max }^{2}$ over a large number of simulations, and calculate the dispersion $D\left( {\omega_{\max } } \right) = \left\langle {\omega_{\max }^{2} } \right\rangle - \left\langle {\omega_{\max } } \right\rangle^{2}$ for different values of the coupling strength $\Omega$ and the reservoir temperatures $T_{1,2}$ (see Fig. 6a). We can see that the behavior of $D\left( {\omega_{\max } } \right)$ changes qualitatively at the point of phase transition ($\Omega^{2} = \Omega_{cr}^{2}$). Near the critical point (CP), the dispersion behaves as $D\left( {\omega_{\max } } \right) \sim \left| {\Omega - \Omega_{cr} } \right|^{\alpha }$. In accordance with the terminology used in the theory of second-order phase transitions^48^, we refer to $\alpha$ as the critical exponent. It can be shown that the dispersion $D\left( {\omega_{\max } } \right)$ and the critical exponent $\alpha$ depend on the ratio of the reservoir temperatures ($T_{2} /T_{1}$) (see Fig. 6b), but do not depend on the absolute values of these temperatures (see “Methods” section).

Note that within a small neighborhood of the transition point, the power law is violated due to the finiteness of the system, a result that also agrees with the theory of second-order phase transitions^48^.

## Conclusions

We demonstrate that in non-Hermitian systems far from equilibrium, a new type of non-Hermitian phase transition takes place. We consider a system of two coupled harmonic oscillators interacting with reservoirs at different temperatures. The difference of the reservoir temperatures moves the system away from thermal equilibrium, resulting in an energy flow through the system. We show that the spectrum of this energy flow is determined by an analog of the $\varphi^{4}$-potential, and exhibits frequency splitting at a CP above which there are two minima in the potential. This spectral splitting at the CP can be associated with a non-equilibrium phase transition. The frequency of the maximum in the spectrum plays the role of an order parameter for this phase transition. We demonstrate that near the CP, fluctuations of the order parameter diverge according to a power law, and we calculate the critical exponent. We show that this depends only on the ratio of the reservoir temperatures, which indicates the non-equilibrium nature of the phase transition.

The non-Hermitian phase transition described here can take place in non-Hermitian systems without an EP. Moreover, this phase transition is not accompanied by qualitative changes in the eigenvalues and eigenstates of the system matrix. This opens the way for the study of non-Hermitian phase transitions in a new class of systems.

## Methods

### Derivation of the system equations

We consider the system of two coupled oscillators interacting with their reservoirs, which are described by infinite sets of oscillators. The Hamiltonian of this system is

10 $$\hat{H} = \hat{H}_{sys} + \hat{H}_{R1} + \hat{H}_{R2} + \hat{V}_{1} + \hat{V}_{2} .$$

Here $\hat{H}_{sys} = \omega_{0} \hat{a}_{1}^{\dag } \hat{a}_{1} + \omega_{0} \hat{a}_{2}^{\dag } \hat{a}_{2} + \Omega \,(\hat{a}_{1}^{\dag } \hat{a}_{2} + \hat{a}_{2}^{\dag } \hat{a}_{1} )$ is a Hamiltonian of the system of two coupled oscillators in the rotating-wave approximation^46^ ($\hbar = 1$), where $\hat{a}_{1,2}$ and $\hat{a}_{1,2}^{\dag }$ are the annihilation and creation operators of the first and second oscillators, obeying the boson commutation relations^46^. $\omega_{0}$ is a frequency of individual oscillators. $\Omega$ is a coupling strength between the oscillators. $\hat{H}_{R1} = \sum\limits_{{k_{1} }} {\omega_{{1k_{1} }} \hat{b}_{{1k_{1} }}^{\dag } \hat{b}_{{1k_{1} }} }$ and $\hat{H}_{R2} = \sum\limits_{{k_{2} }} {\omega_{{2k_{2} }} \hat{b}_{{2k_{2} }}^{\dag } \hat{b}_{{2k_{2} }} }$ are Hamiltonians of the first and second reservoirs, where the summations are over infinite sets of oscillators in the reservoirs. $\hat{b}_{{1k_{1} }}$, $\hat{b}_{{2k_{2} }}$, $\hat{b}_{{1k_{1} }}^{\dag }$, $\hat{b}_{{2k_{2} }}^{\dag }$ are the annihilation and creation operators of the oscillators in the first and second reservoirs. $\omega_{{1k_{1} }}$ and $\omega_{{2k_{2} }}$ are frequencies of these oscillators. $\hat{V}_{1} = \sum\limits_{{k_{1} }} {g_{{1k_{1} }} (\hat{b}_{{1k_{1} }}^{\dag } \hat{a}_{1} + \hat{a}_{1}^{\dag } \hat{b}_{{1k_{1} }} )}$ and $\hat{V}_{2} = \sum\limits_{{k_{2} }} {g_{{2k_{2} }} (\hat{b}_{{2k_{2} }}^{\dag } \hat{a}_{2} + \hat{a}_{2}^{\dag } \hat{b}_{{2k_{2} }} )}$ are Hamiltonians of the interaction between the first/second oscillators and the first/second reservoir, respectively. $g_{{1k_{1} }}$ and $g_{{2k_{2} }}$ are the coupling strength between the first/second oscillator and the $k_{1,2}$-th oscillator in the first/second reservoirs.

Using the Heisenberg equation for operators^43,44^ and then moving from the operators to averages, we obtain the following equations

11 $$\dot{a}_{1} = - i\omega_{0} a_{1} - i\Omega a_{2} - i\sum\limits_{{k_{1} }} {g_{{1k_{1} }} b_{{1k_{1} }} } ,$$

12 $$\dot{a}_{2} = - i\omega_{0} a_{2} - i\Omega a_{1} - i\sum\limits_{{k_{2} }} {g_{{2k_{2} }} b_{{2k_{2} }} } ,$$

13 $$\dot{b}_{{1k_{1} }} = - i\omega_{{1k_{1} }} b_{{1k_{1} }} - ig_{{1k_{1} }} a_{1} ,$$

14 $$\dot{b}_{{2k_{2} }} = - i\omega_{{2k_{2} }} b_{{2k_{2} }} - ig_{{2k_{2} }} a_{2} .$$

Here $a_{1,2} = \left\langle {\hat{a}_{1,2} } \right\rangle$, $b_{{1k_{1} }} = \left\langle {\hat{b}_{{1k_{1} }} } \right\rangle$, $b_{{1k_{2} }} = \left\langle {\hat{b}_{{1k_{2} }} } \right\rangle$ are averages of the respective operators.

Then, we formally integrate the last two equations:

15 $$b_{{1k_{1} }} = b_{{1k_{1} }} (0)e^{{ - i\omega_{{1k_{1} }} t}} - ig_{{1k_{1} }} \int\limits_{0}^{t} {d\tau a_{1} (\tau )e^{{ - i\omega_{{1k_{1} }} (t - \tau )}} } ,$$

16 $$b_{{2k_{2} }} = b_{{2k_{2} }} (0)e^{{ - i\omega_{{2k_{2} }} t}} - ig_{{2k_{2} }} \int\limits_{0}^{t} {d\tau a_{2} (\tau )e^{{ - i\omega_{{2k_{2} }} (t - \tau )}} } .$$

Substituting Eqs. (15) and (16) into Eqs. (11) and (12), respectively, we obtain

17 $$\dot{a}_{1} = - i\omega_{0} a_{1} - i\Omega a_{2} - \sum\limits_{{k_{1} }} {g_{{1k_{1} }}^{2} \int\limits_{0}^{t} {d\tau a_{1} (\tau )e^{{ - i\omega_{{1k_{1} }} (t - \tau )}} } } + f_{1} (t),$$

18 $$\dot{a}_{2} = - i\omega_{0} a_{2} - i\Omega a_{1} - \sum\limits_{{k_{2} }} {g_{{2k_{2} }}^{2} \int\limits_{0}^{t} {d\tau a_{2} (\tau )e^{{ - i\omega_{{2k_{2} }} (t - \tau )}} } } + f_{2} (t),$$

where $f_{1} (t) = - i\sum\limits_{{k_{1} }} {g_{{1k_{1} }} b_{{1k_{1} }} (0)e^{{ - i\omega_{{1k_{1} }} t}} }$ and $f_{2} (t) = - i\sum\limits_{{k_{2} }} {g_{{2k_{2} }} b_{{2k_{2} }} (0)e^{{ - i\omega_{{2k_{2} }} t}} }$ play role of the noise acting on the first and second oscillators^43,44^.

Making a change of variables $\tilde{a}_{1,2} = a_{1,2} e^{{i\omega_{0} t}}$, we obtain the equations for slow variables:

19 $$\dot{\tilde{a}}_{1} = - i\Omega \tilde{a}_{2} - \sum\limits_{{k_{1} }} {g_{{1k_{1} }}^{2} \int\limits_{0}^{t} {d\tau \tilde{a}_{1} (\tau )e^{{ - i(\omega_{{1k_{1} }} - \omega_{0} )(t - \tau )}} } } + \xi_{1} (t),$$

20 $$\dot{\tilde{a}}_{2} = - i\Omega \tilde{a}_{1} - \sum\limits_{{k_{1} }} {g_{{1k_{1} }}^{2} \int\limits_{0}^{t} {d\tau \tilde{a}_{2} (\tau )e^{{ - i(\omega_{{2k_{2} }} - \omega_{0} )(t - \tau )}} } } + \xi_{2} (t),$$

where $$\xi_{1} (t) = - i\sum\limits_{{k_{1} }} {g_{{1k_{1} }} b_{{1k_{1} }} (0)e^{{ - i(\omega_{{1k_{1} }} - \omega_{0} )t}} }$$ and $$\xi_{2} (t) = - i\sum\limits_{{k_{2} }} {g_{{2k_{2} }} b_{{2k_{2} }} (0)e^{{ - i(\omega_{{2k_{2} }} - \omega_{0} )t}} }$$.

Following the Born–Markov approximation^43,44^ and using the Sokhotskii–Plemelj formula^51^, we calculate the integrals in the right parts of Eqs. (19) and (20). As a result, we obtain the equations with the relaxation and noise terms:

21 $$\dot{\tilde{a}}_{1} = - \gamma_{1} \tilde{a}_{1} - i\Omega \tilde{a}_{2} + \xi_{1} (t),$$

22 $$\dot{\tilde{a}}_{2} = - \gamma_{2} \tilde{a}_{2} - i\Omega \tilde{a}_{1} + \xi_{2} (t),$$

where $$\gamma_{1} = \sum\limits_{{k_{1} }} {\pi g_{{1k_{1} }}^{2} \delta (\omega_{{1k_{1} }} - \omega_{0} )}$$ and $$\gamma_{2} = \sum\limits_{{k_{2} }} {\pi g_{{2k_{2} }}^{2} \delta (\omega_{{2k_{2} }} - \omega_{0} )}$$^51^.

Considering that the first and second reservoirs are in thermal equilibrium with temperatures $T_{1}$ and $T_{2}$, respectively, we find the correlation properties of noise:

23 $$\left\langle {\vec{\xi }} \right\rangle = 0,\,\,\,\,\,\left\langle {\vec{\xi }^{*} (t + \tau )\vec{\xi }^{T} (t)} \right\rangle = 2\hat{D}\delta (\tau ),$$

where $\vec{\xi }(t) = \left( {\xi_{1} \left( t \right),\,\,\xi_{2} \left( t \right)} \right)^{T}$ is a vector of noise terms and $$\hat{D} = \left( {\begin{array}{*{20}l} {\gamma_{1} T_{1} } & 0 \\ 0 & {\gamma_{2} T_{2} } \\ \end{array} } \right)$$ is a diffusion matrix^43,44^. Thus, it is seen that in the derived equations, the relaxation and noise terms are related to each other by the fluctuation-dissipative theorem^43,44^.

### Derivation of the expression for the system spectrum

Using the Wiener-Khinchin theorem^46,47^, we calculate the spectrum of a system

24 $$\hat{S}(\omega ) = \frac{1}{2\pi }\int\limits_{ - \infty }^{ + \infty } {d\tau e^{ - i\omega \tau } \left\langle {\vec{a}^{*} (\tau )\vec{a}^{T} } \right\rangle_{st} } .$$

We first calculate the correlator $\left\langle {\vec{a}^{*} (\tau )\vec{a}^{T} } \right\rangle_{st}$ in Eq. (24). To do this, we formally integrate the matrix Eq. (1):

25 $$\vec{a}(t) = \exp (\hat{M}t)\vec{a}(0) + \int\limits_{0}^{t} {d\tau \exp (\hat{M}(t - \tau ))\vec{\xi }(\tau )} .$$

Then, using the quantum regression theorem^43^, we obtain:

26 $$\begin{aligned} \left\langle {\vec{a}^{*} (t + \tau )\vec{a}^{T} (t)} \right\rangle = & \exp (\hat{M}^{*} (t + \tau ))\vec{a}^{*} (0)\vec{a}^{T} (0)\exp (\hat{M}^{T} t) \\ & + \int\limits_{t}^{t + \tau } {d\tau^{\prime}\int\limits_{0}^{t} {d\tau^{\prime\prime}\exp (\hat{M}^{*} (t + \tau - \tau^{\prime}))\left\langle {\vec{\xi }^{*} (\tau^{\prime})\vec{\xi }^{T} (\tau^{\prime\prime})} \right\rangle \exp (\hat{M}^{T} (t - \tau^{\prime\prime}))} } . \\ \end{aligned}$$

All eigenvalues of the matrix $\hat{M}$ have negative real parts. Thus, at times $t \gg \gamma_{1,2}^{ - 1}$, the first term on right side in Eq. (26) tends to zero. Taking into account the expressions for the noise correlators (see Eq. (2)) and considering the case $t \gg \gamma_{1,2}^{ - 1}$, we derive:

27 $$\begin{aligned} \left\langle {\vec{a}^{*} (t + \tau )\vec{a}^{T} (t)} \right\rangle = & 2\int\limits_{0}^{t + \tau } {d\tau^{\prime}\int\limits_{0}^{t} {d\tau^{\prime\prime}\exp (\hat{M}^{*} (t + \tau - \tau^{\prime}))\hat{D}\delta (\tau^{\prime} - \tau^{\prime\prime})\exp (\hat{M}^{T} (t - \tau^{\prime\prime}))} } \\ & = 2\exp (\hat{M}^{*} \tau )\exp (\hat{M}^{*} t)(\int\limits_{0}^{t} {d\tau^{\prime\prime}\exp ( - \hat{M}^{*} \tau^{\prime\prime})\hat{D}\exp ( - \hat{M}^{T} \tau^{\prime\prime})} )\exp (\hat{M}^{T} t). \\ \end{aligned}$$

When $\tau \to + 0$, Eq. (27) takes the form:

28 $$\left\langle {\vec{a}^{*} (t)\,\vec{a}^{T} (t)} \right\rangle = 2\exp (\hat{M}^{*} t)(\int\limits_{0}^{t} {d\tau^{\prime\prime}\exp ( - \hat{M}^{*} \tau^{\prime\prime})\hat{D}\exp ( - \hat{M}^{T} \tau^{\prime\prime})} )\exp (\hat{M}^{T} t).$$

Differentiating both sides of Eq. (28), we obtain:

29 $$\frac{d}{dt}\left\langle {\vec{a}^{*} (t)\vec{a}^{T} (t)} \right\rangle = \hat{M}^{*} \left\langle {\vec{a}^{*} (t)\vec{a}^{T} (t)} \right\rangle + \left\langle {\vec{a}^{*} (t)\vec{a}^{T} (t)} \right\rangle \hat{M}^{T} + 2\hat{D}.$$

In the steady state, $\left\langle {\vec{a}^{*} (t)\vec{a}^{T} (t)} \right\rangle$ is determined by the following equation:

30 $$\hat{M}^{*} \left\langle {\vec{a}^{*} \vec{a}^{T} } \right\rangle_{st} + \left\langle {\vec{a}^{*} \vec{a}^{T} } \right\rangle_{st} \hat{M}^{T} + 2\hat{D} = 0.$$

To calculate the correlator $\left\langle {\vec{a}^{*} (\tau )\,\vec{a}^{T} } \right\rangle_{st}$, which is determined as $\left\langle {\vec{a}^{*} (\tau )\,\vec{a}^{T} } \right\rangle_{st} = \left\langle {\vec{a}^{*} (t + \tau )\,\vec{a}^{T} (t)} \right\rangle_{t \to \infty }$, we use Eq. (28):

31 $$\left\langle {\vec{a}^{*} (\tau )\,\vec{a}^{T} } \right\rangle_{st} = \exp (\hat{M}^{*} \tau )\left\langle {\vec{a}^{*} \vec{a}^{T} } \right\rangle_{st} ,\tau > 0.$$

The expression in Eq. (31) holds true when $\tau > 0$. To use the Wiener–Khinchin theorem^46,47^ we need to calculate the correlator at $\tau < 0$. This correlator is calculated in the same way, as follows:

32 $$\left\langle {\vec{a}^{*} (\tau )\,\vec{a}^{T} } \right\rangle_{st} = \left\langle {\vec{a}^{*} \vec{a}^{T} } \right\rangle_{st} \exp ( - \hat{M}^{T} \tau ),\tau < 0.$$

Thus, the spectrum can be expressed as:

33 $$\begin{aligned} \hat{S}(\omega ) = & \frac{1}{2\pi }\int\limits_{ - \infty }^{ + \infty } {d\tau e^{ - i\omega \tau } \left\langle {\vec{a}^{*} (\tau )\,\vec{a}^{T} } \right\rangle_{st} } = \frac{1}{2\pi }\left\langle {\vec{a}^{*} \vec{a}^{T} } \right\rangle_{{{\text{st}}}} \int\limits_{ - \infty }^{0} {d\tau \exp (( - \hat{M}^{T} - i\omega \hat{I})\tau )} \\ & + \frac{1}{2\pi }\int\limits_{0}^{\infty } {d\tau \exp ((\hat{M}^{*} - i\omega \hat{I})\tau )} \left\langle {\vec{a}^{*} \vec{a}^{T} } \right\rangle_{{{\text{st}}}} \\ = & \frac{1}{2\pi }\int\limits_{0}^{ + \infty } {d\tau (\left\langle {\vec{a}^{*} \vec{a}^{T} } \right\rangle_{{{\text{st}}}} \exp ((\hat{M}^{T} + i\omega \hat{I})\tau )} + \exp ((\hat{M}^{*} - i\omega \hat{I})\tau )\left\langle {\vec{a}^{*} \vec{a}^{T} } \right\rangle_{{{\text{st}}}} ) \\ = & - \frac{1}{2\pi }\left( {\left\langle {\vec{a}^{*} \vec{a}^{T} } \right\rangle_{{{\text{st}}}} (\hat{M}^{T} + i\omega \hat{I})^{ - 1} + (\hat{M}^{*} - i\omega \hat{I})^{ - 1} \left\langle {\vec{a}^{*} \vec{a}^{T} } \right\rangle_{{{\text{st}}}} } \right). \\ \end{aligned}$$

By multiplying the spectrum matrix on the left and right by the respective matrices and taking into account Eqs. (31) and (32), we obtain:

34 $$\begin{aligned} (\hat{M}^{*} - i\omega \hat{I})\,\hat{S}(\omega )(\hat{M}^{T} + i\omega \hat{I}) = & - \frac{1}{2\pi }((\hat{M}^{*} - i\omega \hat{I})\left\langle {\vec{a}^{*} \vec{a}^{T} } \right\rangle_{{{\text{st}}}} + \left\langle {\vec{a}^{*} \vec{a}^{T} } \right\rangle_{{{\text{st}}}} (\hat{M}^{T} + i\omega \hat{I})) \\ = & - \frac{1}{2\pi }(\hat{M}^{*} \left\langle {\vec{a}^{*} \vec{a}^{T} } \right\rangle_{{{\text{st}}}} + \left\langle {\vec{a}^{*} \vec{a}^{T} } \right\rangle_{{{\text{st}}}} \hat{M}^{T} ) =\hat{D}/\pi . \\ \end{aligned}$$

Finally, we obtain an expression for the spectrum matrix:

35 $$\hat{S}(\omega ) = \frac{1}{\pi }(\hat{M}^{*} - i\omega \hat{I})^{ - 1} \hat{D}(\hat{M}^{T} + i\omega \hat{I})^{ - 1} .$$

By substituting into Eq. (35) the expressions for the matrices $\hat{M}$ and $\hat{D}$ from the main text, we obtain:

36 $$\hat{S}(\omega ) = \frac{1/\pi }{{\Phi (\omega )}}\left( {\begin{array}{*{20}c} {\gamma_{1} T_{1} (\gamma_{2}^{2} + \omega^{2} ) + \gamma_{2} T_{2} \,\Omega^{2} } & {i\,\Omega \left( {\gamma_{2} T_{2} (\gamma_{1} + i\omega ) - \gamma_{1} T_{1} (\gamma_{2} - i\omega )} \right)} \\ {i\Omega \,\left( {\gamma_{1} T_{1} (\gamma_{2} + i\omega ) - \gamma_{2} T_{2} (\gamma_{1} - i\omega )} \right)} & {\gamma_{2} T_{2} (\gamma_{1}^{2} + \omega^{2} ) + \gamma_{1} T_{1} \,\Omega^{2} } \\ \end{array} } \right),$$

where $$\Phi (\omega ) = (\gamma_{1} \gamma_{2} + \Omega^{2} )^{2} - 2(\Omega^{2} - \Omega_{cr}^{2} )\omega^{2} + \omega^{4}$$ and $$\Omega_{cr}^{2} = \left( {\gamma_{1}^{2} + \gamma_{2}^{2} } \right)/2$$.

The diagonal elements of $\hat{S}(\omega )$ define the spectra of the first and second oscillators, and the non-diagonal elements are complex quantities. The real parts of the non-diagonal elements are equal to each other, and define the spectrum of interaction between the oscillators, while the imaginary parts of the non-diagonal elements differ from each other in sign and define the spectra of the energy flow from the first oscillator to the second and vice versa.

Note that $\omega$ is the difference between the oscillation frequency and the frequency of a single oscillator $\omega_{0}$ (we consider that the frequencies of oscillators are equal to each other).

### Dependence of the dispersion of the order parameter on the ratio of the reservoir temperatures

Numerical simulation of Eq. (1) shows that the spectra of the oscillators, the spectra of the interaction and the energy flow between the oscillators, and the dispersion of the frequency of the maximum in the spectrum ($\omega_{\max }$) of energy flow $J\left( \omega \right)$ depend on the ratio of the reservoir temperatures rather than their absolute values. To ascertain the mechanism of this dependence, we analyze the behavior of the system with noise, as follows:

37 $$\frac{d}{dt}\left( {\begin{array}{*{20}c} {a_{1} } \\ {a_{2} } \\ \end{array} } \right) = \left( {\begin{array}{*{20}c} { - \gamma_{1} } & { - i\Omega } \\ { - i\Omega } & { - \gamma_{2} } \\ \end{array} } \right)\left( {\begin{array}{*{20}c} {a_{1} } \\ {a_{2} } \\ \end{array} } \right) + \left( {\begin{array}{*{20}c} {\xi_{1} (t)} \\ {\xi_{2} (t)} \\ \end{array} } \right) = \hat{M}\vec{a} + \vec{\xi }(t),$$

where $$\vec{a} = \left( {\begin{array}{*{20}c} {a_{1} } & {a_{2} } \\ \end{array} } \right)^{T}$$ is a vector of the amplitudes of the first and second oscillators, respectively; $\Omega$ is the coupling strength between the oscillators; $\gamma_{1,2}$ are the relaxation rates of the oscillators; and $$\vec{\xi } = \left( {\begin{array}{*{20}c} {\xi_{1} } & {\xi_{2} } \\ \end{array} } \right)^{T}$$ is a noise term that obeys the following conditions

38 $$\left\langle {\vec{\xi }} \right\rangle = 0,\,\,\,\,\,\left\langle {\vec{\xi }^{*} (t + \tau )\vec{\xi }^{T} (t)} \right\rangle = 2\hat{D}\delta (\tau ),$$

where $$\hat{D} = \left( {\begin{array}{*{20}c} {\gamma_{1} T_{1} } & 0 \\ 0 & {\gamma_{2} T_{2} } \\ \end{array} } \right)$$ is a diffusion matrix.

In the stationary state, the amplitudes $a_{1,2}$ are nonzero only when at least one of the reservoir temperatures is nonzero. Without loss of generality, we assume that $T_{2} \ne 0$. In this case, we can make changes to the variables $\tilde{a}_{1,2} = a_{1,2} /\sqrt {T_{2} }$ and $\tilde{\xi }_{1,2} = \xi_{1,2} /\sqrt {T_{2} }$. In these new variables, the Eq. (37) can be rewritten as:

39 $$\frac{d}{dt}\tilde{\vec{a}} = \hat{M}\tilde{\vec{a}} + \tilde{\vec{\xi }}(t).$$

The redefined noise terms obey the conditions

40 $$\left\langle {\tilde{\vec{\xi }}} \right\rangle = 0,\,\,\,\,\,\left\langle {\tilde{\vec{\xi }}^{*} (t + \tau )\tilde{\vec{\xi }}^{T} (t)} \right\rangle = 2\tilde{\hat{D}}\delta (\tau ),$$

where $$\tilde{\hat{D}} = \left( {\begin{array}{*{20}c} {\gamma_{1} \,T_{1} /T_{2} } & 0 \\ 0 & {\gamma_{2} } \\ \end{array} } \right)$$ is a redefined diffusion matrix. It is important to note that $\tilde{\hat{D}}$, and consequently the amplitudes $$\tilde{\vec{a}} = \left( {\begin{array}{*{20}c} {\tilde{a}_{1} } & {\tilde{a}_{2} } \\ \end{array} } \right)^{T}$$, depend only on the ratio of the reservoir temperatures. As a result, the spectrum matrix $\tilde{\hat{S}}(\omega )$ calculated by Eq. (39) depends on the ratio of the reservoir temperatures (where the method of calculation is the same as for Eq. (37)). The spectrum matrix $\hat{S}\left( \omega \right)$ is determined by $\tilde{\hat{S}}(\omega )$ as (see the definition of $\hat{S}\left( \omega \right)$ in Eq. (3)):

41 $$\hat{S}\left( \omega \right) = T_{2} \,\tilde{\hat{S}}\left( \omega \right).$$

Thus, the form of spectrum $\hat{S}\left( \omega \right)$ depends only on the ratio of the reservoir temperatures, while the absolute value of the temperature $T_{2}$ is determined only by the amplitude of the spectrum. The same conclusion holds true for a spectrum calculated based on a single simulation of Eq. (37). As a result, the frequency of the maximum in the spectrum ($\omega_{\max }$) calculated for this simulation also depends only on the ratio of the reservoir temperatures. Since the dispersion of the frequency $D\left( {\omega_{\max } } \right) = \left\langle {\omega_{\max }^{2} } \right\rangle - \left\langle {\omega_{\max } } \right\rangle^{2}$ is calculated by averaging $\omega_{\max }$ and $\omega_{\max }^{2}$ over a large number of simulations, then $D\left( {\omega_{\max } } \right)$ depends only on the ratio of the reservoir temperatures.

### Expressions for $\Omega_{1}^{split}$ and $\Omega_{2}^{split}$

The coupling strengths $\Omega_{1}^{split}$ and $\Omega_{2}^{split}$, at which the splitting in the fluctuation spectra of the first and second oscillators occurs, are given as

$$\left( {\Omega_{1}^{split} } \right)^{2} = \frac{{\gamma_{1}^{2} \gamma_{2} T_{2} + \gamma_{2}^{3} T_{2} - 2\gamma_{1}^{2} \gamma_{2} T_{1} - 2\gamma_{1} \gamma_{2}^{2} T_{1} }}{{2\left( {\gamma_{1} T_{1} + 2\gamma_{2} T_{2} } \right)}} + \frac{{\sqrt {4\gamma_{1} \gamma_{2}^{4} T_{1} \left( {\gamma_{1} T_{1} + 2\gamma_{2} T_{2} } \right) + \left( {\gamma_{2} \left( {\gamma_{1}^{2} + \gamma_{2}^{2} } \right)T_{2} - 2\gamma_{1} \gamma_{2} \left( {\gamma_{1} + \gamma_{2} } \right)T_{1} } \right)^{2} } }}{{2\left( {\gamma_{1} T_{1} + 2\gamma_{2} T_{2} } \right)}}{,}$$

$$\left( {\Omega_{2}^{split} } \right)^{2} = \frac{{\gamma_{2}^{2} \gamma_{1} T_{1} + \gamma_{1}^{3} T_{1} - 2\gamma_{1}^{2} \gamma_{2} T_{2} - 2\gamma_{1} \gamma_{2}^{2} T_{2} }}{{2\left( {\gamma_{2} T_{2} + 2\gamma_{1} T_{1} } \right)}} + \frac{{\sqrt {4\gamma_{2} \gamma_{1}^{4} T_{2} \left( {\gamma_{2} T_{2} + 2\gamma_{1} T_{1} } \right) + \left( {\gamma_{1} \left( {\gamma_{1}^{2} + \gamma_{2}^{2} } \right)T_{1} - 2\gamma_{1} \gamma_{2} \left( {\gamma_{1} + \gamma_{2} } \right)T_{2} } \right)^{2} } }}{{2\left( {\gamma_{2} T_{2} + 2\gamma_{1} T_{1} } \right)}}.$$
